# Contribution of the LIM Domain and Nebulin-Repeats to the Interaction of Lasp-2 with Actin Filaments and Focal Adhesions

**DOI:** 10.1371/journal.pone.0007530

**Published:** 2009-10-23

**Authors:** Hiroyuki Nakagawa, Hiroshi Suzuki, Satoshi Machida, Junko Suzuki, Kazuyo Ohashi, Mingyue Jin, Shigeaki Miyamoto, Asako G. Terasaki

**Affiliations:** 1 Division of Biology, Faculty of Science, Fukuoka University, Fukuoka, Japan; 2 Graduate School of Advanced Integration Science, Chiba University, Chiba, Japan; 3 Department of Biology, Chiba University, Chiba, Japan; 4 Department of Bioscience and Bioinformatics, Kyushu Institute of Technology, Iizuka, Fukuoka, Japan; University of Birmingham, United Kingdom

## Abstract

Lasp-2 binds to actin filaments and concentrates in the actin bundles of filopodia and lamellipodia in neural cells and focal adhesions in fibroblastic cells. Lasp-2 has three structural regions: a LIM domain, a nebulin-repeat region, and an SH3 domain; however, the region(s) responsible for its interactions with actin filaments and focal adhesions are still unclear. In this study, we revealed that the N-terminal fragment from the LIM domain to the first nebulin-repeat module (LIM-n1) retained actin-binding activity and showed a similar subcellular localization to full-length lasp-2 in neural cells. The LIM domain fragment did not interact with actin filaments or localize to actin filament bundles. In contrast, LIM-n1 showed a clear subcellular localization to filopodial actin bundles. Although truncation of the LIM domain caused the loss of F-actin binding activity and the accumulation of filopodial actin bundles, these truncated fragments localized to focal adhesions. These results suggest that lasp-2 interactions with actin filaments are mediated through the cooperation of the LIM domain and the first nebulin-repeat module *in vitro* and *in vivo*. Actin filament binding activity may be a major contributor to the subcellular localization of lasp-2 to filopodia but is not crucial for lasp-2 recruitment to focal adhesions.

## Introduction

During locomotion on a substrate, an attached cell extends lamellipodia and filopodia, which are induced via the Rho family small G-proteins Rac and Cdc42, respectively [1]. In lamellipodia, the dynamics of actin filaments and their accompanying accessory proteins have been described in detail [2]. Although filopodia are the projections of actin filament bundles from lamellipodia, some specific proteins are localized to actin bundles in filopodia [1], [3]. Formin family molecules are thought to stimulate the polymerization of actin filaments at filopodia tips instead of Arp2/3 complex, a major nucleator of actin polymerization in lamellipodia [3]–[6]. Fascin has been proposed to cross-link elongating actin filaments into actin bundles in lamellipodia to produce filopodia [7], [8]. Under light microscopy, filopodial actin bundles are observed to run across lamellipodia [8], [9]. The dynamics of these proteins have been observed during filopodia formation, but their roles in the organization of actin bundles in filopodia are still under discussion [5]–[8].

Recently, we reported a novel F-actin-binding protein, lasp-2, which was identified using a proteomic approach in chicken brain extracts [10]. Li and Trueb characterized lasp-2 independently as a novel zyxin-binding protein, namely LIM-nebulette [11]. Lasp-2 is a molecule related to lasp-1, which has been identified as a human gene product amplified in breast carcinoma [12], [13]. As in lasp-1, lasp-2 contains three structural regions: a LIM domain at the N-terminus, followed by a tandem plural nebulin-repeat region and an SH3 domain in the C-terminal region [10], [11], [14], [15]. Although lasp-1 is a *LASP1* gene product in mammals and aves, lasp-2 is a splicing variant from the *LASP2/NEBL* gene, which concurrently encodes nebulette, an actin-binding protein expressed specifically in cardiac muscle [14], [15]. The mRNA of vertebrate lasp-2 is constituted from seven exons of the *LASP2/NEBL* gene [14], [16]. Both lasp-1 and lasp-2 bind to F-actin *in vitro* and colocalize at filopodial actin bundles and focal adhesions in several established cell lines [8], [10], [11], [17]. In addition, Zieseniss et al. recently identified lasp-2 as a Z-disc associated protein in striated muscle [18]. In contrast to the ubiquitous expression of lasp-1, lasp-2 is mainly detected in the brain [10]. Using EGFP fused lasp-1 and -2, their turnover rates on actin filaments were found to not be closely correlated with the reorganization of the actin cytoskeleton during the extension of filopodia and lamellipodia [8].

The LIM and SH3 domains have been recognized in both lasp-1 and lasp-2. These domains play roles in various signaling pathways, but their functions in lasp-1 and lasp-2 have not been fully analyzed. The LIM domain is characterized as a tandem zinc-finger structure in many proteins, which participate in diverse cellular functions in gene expression, cell adhesion, and signal transduction [19]. The LIM domains of several proteins have been reported to interact with F-actin. Khurana et al. (2002) showed that the LIM domain of LimC, a *Dictyostelium* homolog of the mammalian cysteine-rich protein (CRP) family, binds to F-actin [20]. Mammalian CRPs also interact with F-actin and localize to actin stress fibers in cultured cells [21], [22]. The SH3 domain has been identified in many molecules that regulate the dynamics of the actin cytoskeleton [23], [24]. In such molecules, the SH3 domain provides an interface for protein-protein interactions involved in the formation of large protein complexes, but the actin-binding activity of the SH3 domain has not been reported.

A recombinant lasp-1 fragment containing the LIM domain and two nebulin repeats has been shown to bind to actin molecules [25]. The domain responsible for the binding, however, has not been analyzed in detail. The SH3 domains of both lasp-1 and lasp-2 have been reported to regulate their subcellular localization to focal adhesions [11], [26]. Recently, Panaviene and Moncman (2007) suggested the importance of the linker sequence connecting the nebulin-repeat region to the SH3 domain for the recruitment of lasp-1 and lasp-2 to focal adhesions [27]. Most previous studies on the subcellular localization of lasp-1 and lasp-2 have focused on the role of the SH3 domain, rather than on their actin-binding activity. The domain structure of lasp-2 is similar to that of lasp-1 except for the linker sequence, in which the third nebulin-repeat of lasp-2 exists between the second nebulin-repeat and SH3 domain [11], [14]. Since the LIM domain, nebulin-repeat region, and SH3 domain of lasp-1 have significant homology to those of lasp-2 [10], the actin-binding region is probably localized in these domains. To clarify the region of lasp-2 responsible for actin-binding and its subcellular localization, we subjected lasp-2 fragments to an F-actin-binding assay and investigated its exogenous expression in cultured cells.

## Results

**Figure 1:**
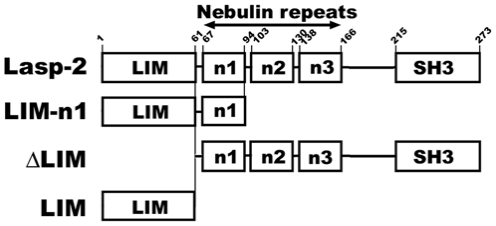
A schematic representation of the domain structure of avian lasp-2 and its fragments. LIM and SH3 indicate the LIM and Src homology 3 domains, respectively. The number of nebulin-repeats is shown by n1 to n3. The LIM-n1 fragment contains the LIM domain and first nebulin-repeat. ΔLIM is a deletion fragment of the LIM domain. The LIM fragment contains only the LIM domain. The numbers indicate the positions of the corresponding amino acid residues.

**Figure 2:**
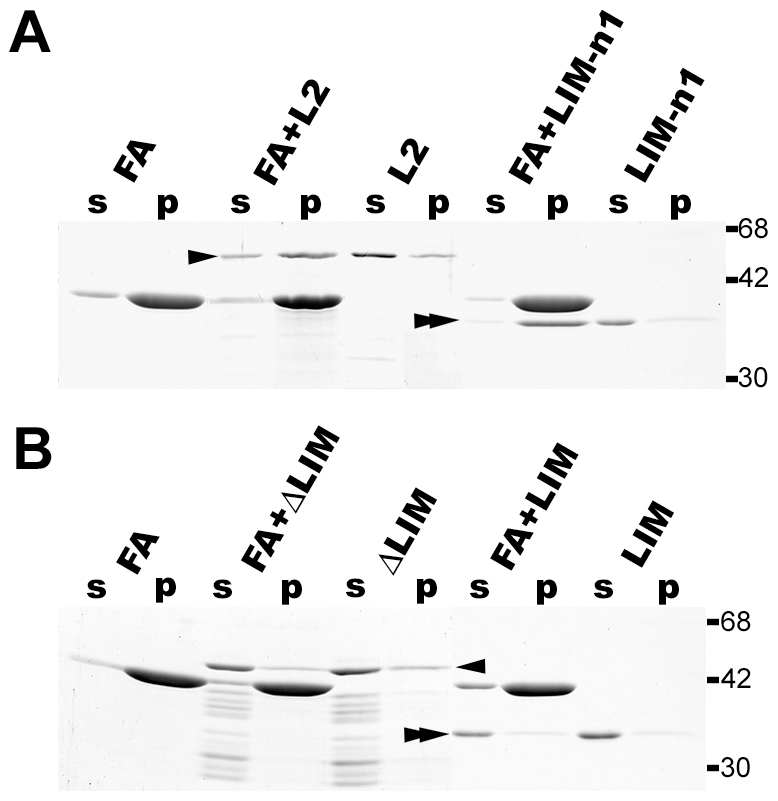
F-actin-binding activity of recombinant lasp-2 and its fragments in the co-sedimentation assay. 0.2 mg/ml G-actin was polymerized with GST-lasp-2 (L2), GST-LIM-n1 (LIM-n1), GST-ΔLIM (ΔLIM), or GST-LIM (LIM). The molar ratio of GST-fused fragment to G-actin was set at 1/5. GST-lasp-2 (arrowhead in A) and GST-LIM-n1 (double-arrowhead in A) were co-precipitated with actin filaments, but GST-ΔLIM (arrowhead in B) and GST-LIM (double-arrowhead in B) were not. FA indicates a control experiment using F-actin without the recombinant peptides. The precipitants (p) were separated from the supernatant (s) by ultracentrifugation. The arrowheads and double-arrowheads indicate bands of recombinant GST fused proteins with the predicted molecular mass. The mobilities of molecular mass markers are listed on the right of the gel images in kilodaltons.

To determine the region responsible for the actin-binding activity of the lasp-2 molecule, we prepared recombinant fragments of lasp-2 in order to subject them to a co-sedimentation assay with F-actin (Figure 1). The GST fragment was not precipitated with F-actin under these experimental conditions (data not shown) [10]. As reported previously, the full-length lasp-2 molecule was precipitated with F-actin (Figure 2A, arrowhead). The N-terminal fragment covering the LIM domain to the first nebulin-repeat (LIM-n1) was precipitated with F-actin (Figure 2A, double-arrowhead). To clarify the contributions of the LIM domain and the first nebulin-repeat to the actin-binding activity of lasp-2, we subjected a truncated fragment of LIM (ΔLIM) and a full-length LIM fragment (LIM) to the co-sedimentation assay (Figure 2B). Although ΔLIM contained the nebulin-repeats that have been proposed to be involved in the interaction of F-actin with lasp-1, most of the fragment was retained in the supernatant rather than being co-precipitated with F-actin (Figure 2B, arrowhead). LIM did not show any interaction with F-actin (Figure 2B, double-arrowhead). To clarify the possibility that F-actin interacts with the junction between the LIM domain and the first nebulin-repeat, a truncated fragment of the exon 1 coding-region of lasp-2 (Δe1), which was reduced by 23 AA at the N-terminal of the LIM domain (Supporting Information Figure S1A), was applied to the co-sedimentation assay (Supporting Information Figure S1B and Text S1). Δe1 was not precipitated with F-actin (Supporting Information Figure S1B, arrowhead). According to these results, the LIM domain region participates in the interaction of the lasp-2 molecule with F-actin together with the first nebulin-repeat.

**Figure 3:**
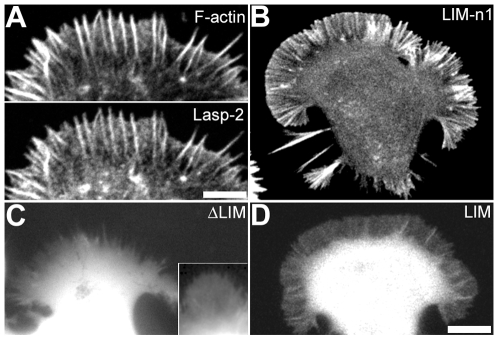
Localization of EGFP-tagged lasp-2 fragments in NG108-15 cells. Cells transfected with EGFP-lasp-2 (A), EGFP-LIM-n1 (B), EGFP-ΔLIM (C), or EGFP-LIM (D) are shown as indicated. The inset in C shows widely distributed lamellipodial extension. Actin filaments were visualized by staining with rhodamine-phalloidin (F-actin in A). The bars in A and D represent 5 and 10 µm, respectively.

**Figure 4:**
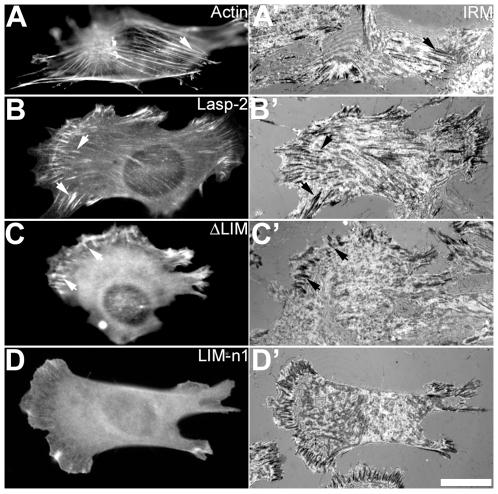
Localization of EGFP-tagged lasp-2 fragments in C2C12 cells. Cells transfected with EGFP-actin (A), EGFP-lasp-2 (B), EGFP-ΔLIM (C), or EGFP-LIM-n1 (D) are shown as indicated. Corresponding images of interference refraction microscopy (IRM) are shown as A′ to D′. The block arrows indicate the position of focal contacts visualized by IRM, and the corresponding areas in the fluorescence images are indicated by white arrows. The bar in D′ represents 10 µm.

To confirm the relationship between the actin-binding activity and subcellular localization of lasp-2, we expressed the fragments examined in the co-sedimentation assay with F-actin in NG108-15 neuroblastoma cells (Figure 3). All fragments were tagged with EGFP instead of GST to the N-terminal of the fragments (see Figure 1). EGFP-lasp-2 has been reported to show similar localization to endogenous lasp-2 molecules [18]. As is the case in neural growth cones, NG108-15 cells do not develop stress fibers or focal adhesions of a detectable size under a light microscope [8], [28]. Full-length lasp-2 localized to actin bundles in extended lamellipodia as described previously [10] (Figure 3A). These bundles projected from the tips of lamellipodia as filopodia. LIM-n1 localized to the bundles in a similar manner to full-length lasp-2 (Figure 3B). LIM and ΔLIM, both fragments with defective F-actin binding activity *in vitro*, were scattered in the cytoplasm and did not show any specific distribution in lamellipodia (Figure 3C, D). As found for ΔLIM, Δe1 showed diffuse distribution in NG108-15 cells (Supporting Information Figure S1C). These results provide evidence that F-actin binding activity is essential for the localization of lasp-2 to filopodial actin bundles. The SH3 domains of lasp-1 and lasp-2 have been shown to regulate their localization to focal adhesions in fibroblastic cells [11], [26]. Our observations, however, indicated that the SH3 domain does not play a crucial role in the localization of lasp-2 to filopodial actin bundles in lamellipodia extending from neuronal cells. This inconsistency implies that the element of the lasp-2 molecule responsible for its subcellular localization differs between neuronal and non-neuronal cells. The focal adhesions of C2C12 myoblasts have been well characterized, and full-length lasp-2 localized to focal adhesions in these cells as visualized by interference reflection microscopy (Figure 4B, B′). Although ΔLIM showed defective F-actin binding, it accumulated to focal adhesions similar to full-length lasp-2 (Figure 4C, C′). LIM-n1, which retained F-actin binding activity, was distributed sparsely in the cytoplasm of C2C12 cells, developing many focal adhesions (Figure 4D, D′). These results show that the F-actin binding activity of lasp-2 does not primarily contribute to its recruitment to focal adhesions.

Among the lasp-2 fragments fused at their N-terminal with GST or GFP, only LIM showed neither F-actin binding nor a specific subcellular localization. The remaining fragments demonstrated at least one of these activities. These results indicated that the N-terminal fusion of GST or GFP does not interrupt the functions of lasp-2 fragments.

## Discussion

**Figure 5:**
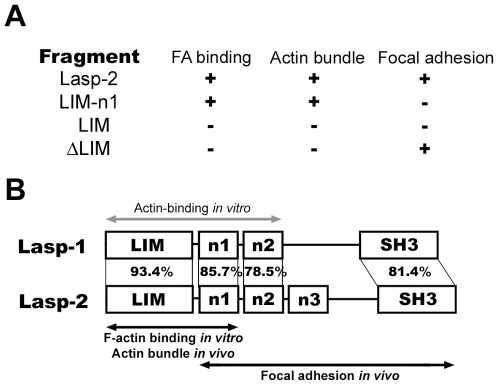
The predicted functions of structural domains in lasp-2. The results of this study are summarized in A. (+) indicates interaction with actin filaments, localization to actin bundles in NG108-15 cells, or focal adhesion in C2C12 cells. (−) indicates negative results compared to (+). The identities of the amino acid sequences of the LIM domain, first nebulin-repeat region, and SH3 domain between lasp-1 and lasp-2 are shown in B. The grey double-headed arrow above lasp-1 indicates the region containing actin-binding activity as reported by Schreiber et al. (1998). The black double-headed arrows under lasp-2 show the regions responsible for localization to focal adhesions and interaction with F-actin *in vitro* and *in vivo*.

As summarized in Figure 5, we showed that both the LIM domain and first nebulin-repeat of lasp-2 are necessary for the interaction of lasp-2 with F-actin *in vitro* and its subcellular localization to filopodial actin bundles in lamellipodia extending from neural cells. It has been reported that actin molecules bind to an N-terminal fragment of lasp-1 composed of the LIM domain and a tandem twin nebulin-repeat [25]. In addition, various nebulin fragments containing plural nebulin-repeats from the nebulin superfamily (nebulin, nebulette, and N-RAP) have been shown to associate with F-actin [29]–[31]. From these results, it has been proposed that the region containing multiple nebulin-repeats participates in the association of lasp-1 and lasp-2 with F-actin [10]. Here, however, we observed that the LIM-n1 fragment, which contained only a single nebulin-repeat, was able to bind to F-actin in the same way as full-length lasp-2. This implies that the first nebulin-repeat has sufficient affinity to anchor the lasp-2 molecule to F-actin. Accordingly, the deletion of the LIM domain in lasp-2 was not expected to affect the interaction between F-actin and lasp-2. The ΔLIM and Δe1fragments, however, showed decreased actin-binding activity. This indicates that the LIM domain participates in the interaction of lasp-2 with F-actin in addition to the first nebulin-repeat module. We also analyzed the actin-binding activity of the LIM domain alone, but the LIM fragment of lasp-2 did not associate with F-actin. Our present results indicate that these domains in the lasp-2 molecule cooperate to achieve a structure that is able to interact with F-actin. Lasp-2 has been shown to form F-actin bundles *in vitro* at a high molar ratio to actin of over 1/2 [18]. Considering this observation and our results, it is suggested that a plural weak F-actin binding region is distributed over the LIM-n1 fragment. Closer analysis of the three-dimensional structure of the LIM-n1 fragment would shed further light on the interaction of lasp-2 with F-actin.

It has been proposed that the SH3 domain and the adjacent linker sequence in the C-terminal of lasp-2 are necessary for its recruitment to focal adhesions [11], [27]. Our results indicate that these regions are essential for the localization of lasp-2 to focal adhesions but not to actin bundles in lamellipodia extending from neural cells. Lasp-2 is considered to be recruited to focal adhesions via the interaction of its SH3 domain with focal adhesion specific molecule(s). In lamellipodia, the localization of truncated lasp-2 fragments to actin bundles seems to be compatible with their actin-binding ability *in vitro*. These results suggest that lasp-2 attaches directly to actin filaments without any linker molecules in lamellipodia. Although lasp-2 binds with F-actin, it accumulates mainly in focal adhesions rather than at stress fibers in non-neural cells as previously reported [32] (Figure 3). In mature myofibrils, lasp-2 has been shown to localize to Z-discs but not to thin filaments [18]. These observations indicate that lasp-2 competes with actin binding proteins for site(s) on actin filaments *in vivo*. It is known that the compositions of actin binding proteins are different between stress fibers and filopodial actin bundles [5], [33]. Biochemical analysis of the competition between lasp-2 and actin binding proteins accumulating at stress fibers, such as tropomyosin and myosin II, would provide information on the interactions of lasp-2 with actin filaments.

The C-terminal region containing the SH3 domain and the linker sequence of lasp-1 have been shown to be nonessential for the translocation of lasp-1 from focal adhesions to the actin-rich leading edge in migrating fibroblasts [26]. Schreiber et al. showed the interaction of a lasp-1 fragment with a defective SH3 domain with actin [25]. Considering this previous report and the marked homology between lasp-1 and -2 in the LIM domain and n1 regions (93.7% and 85.7%, respectively) (Figure 5), these regions may be conserved for F-actin binding in both molecules. It has been reported that various invertebrates have genes encoding proteins homologous to vertebrate lasps [16]. Of these invertebrate lasps, the recombinant ascidian lasp molecule is indeed able to bind to F-actin *in vitro* [16]. The LIM domain and first nebulin-repeat of ascidian lasp showed high similarity to those of vertebrates, but other nebulin-repeats and linker sequences showed less homology.

Using NG108-15 cells, which extend large lamellipodia with many actin bundles, we were able to compare the distribution of lasp-2 fragments with that of the bundles in motile regions. In neural cells, however, the cell-to-substrate adhesion structure has not yet been well characterized. Thus, we expressed the fragments of lasp-2 in C2C12 myoblasts to observe their subcellular localization (Figure 4). Actin filament binding activity was not crucial for the recruitment of lasp-2 molecules to focal adhesions. These results suggest that the subcellular distribution of lasp-2 is regulated through at least two regions of the molecule; one is LIM-n1, and the other the remainder of the C-terminal. The LIM-n1 region is suggested to be responsible for the localization of lasp-2 to filopodial actin bundles though its interaction with actin filaments [8], and the remaining region is considered to be responsible for its localization to focal adhesions through interactions with focal adhesion component(s), such as zyxin and α-actinin [11], [27]. Recently, l-lasp, one of the lasp isoforms in *Drosophila*, has been shown to link *Oskar* product via its SH3 domain to the actin cytoskeleton in early embryos [34]. In vertebrate cells, lasp-1 and -2 may connect signal molecule(s) to the actin cytoskeleton during cell locomotion.

The actin-binding activity of lasp-1 is modulated via phosphorylation mediated by cGMP- and cAMP-dependent protein kinases and Abl [17], [26]. The consensus amino acid sequences of several phosphorylation sites in lasp-1 are preserved in lasp-2 [10]. Further investigation regarding the phosphorylation of lasp-2 is required. Targeted disruption of the *Lasp-1* gene did not provide a significant phenotype in genetically modified mice [35]. This result implies a redundancy of lasp-2 to lasp-1. Biochemical and cell biological analysis of lasp-2 in these mice would shed light on the regulation of lasp-2 functions.

## Materials and Methods

### Expression constructs

pGEX-lasp-2 and pEGFP-lasp-2 were reported previously [8], [10]. To construct the N-terminal-tagged molecule, a cDNA fragment encoding lasp-2 or its fragment was inserted into a cloning site following a GST or EGFP sequence. The cDNA of the lasp-2 fragments were amplified by PCR from the plasmids using the following primer sets, which are shown in the 5′ to 3′ direction: LIM-n1: CGCGGATCCACCATGAACCCCCAG and CCGGAATTCGAAGGACTGCTTTGGGTAATG, LIM: CGCGGATCCACCATGAACCCCCAG and CCGGAATTCGAACCCTCTTCCTTTGC, ΔLIM: CGCGGATCCTCCTTCACTACAGTGGC and GGGGAATTCGGACTAGAGACAAAAATTAG.

### F-actin co-sedimentation assay

Bacterially-expressed recombinant proteins fused with GST were purified with a glutathione-Sepharose 4B column (Amersham Biosciences). Actin was prepared from rabbit back muscle according to the method of Spudich and Watt (1971) [36]. The actin-binding activity of the recombinant proteins was analyzed by the following method as described previously [10]. We treated with all animals used for the experiments according to the guidelines of Chiba University Institutional Animal Care and Use Committee. Recombinant proteins were dialyzed against F-actin buffer (20 mM Na-PO_4_ and 0.1 M KCl, pH 7.2). After centrifugation at 100,000 g for one hour, each of the supernatants was mixed with 0.2 mg/ml G-actin in F-actin buffer containing 5 mM MgCl_2_ and 0.2 mM ATP. After two hours incubation at room temperature, the mixtures were centrifuged at 100,000 g for one hour. The supernatants and the pellets were subsequently analyzed by SDS–PAGE.

### Fluorescence microscopy

NG108-15 neuroblastomas were cultured in DMEM with 10% FetalClone III (HyClone) and penicillin/streptomycin (Sigma-Aldrich) at 37°C as described previously [10], [28]. Cover glasses were sequentially treated with poly-L-lysine (Sigma-Aldrich) and laminin (Sigma-Aldrich) just prior to use. pEGFP constructs were transfected into NG108-15 cells using SuperFect (QIAGEN) in accordance with the manufacturer's instructions. Transfected cells were cultured in an open heating chamber as described previously [8], [37]. The chamber was mounted on a confocal laser microscope (LSM510, Carl Zeiss) to obtain a fluorescence image. To visualize actin filament distribution, the cells were stained with rhodamine-phalloidin as described elsewhere [9]. The contrast of the acquired images was adjusted with ImageJ v1.32 (http://rsb.info.nih.gov/ij/) and arranged in the figures using Adobe Photoshop 6.0 (Adobe Systems).

### Interference reflection microscopy

C2C12 mouse skeletal myoblasts were cultured in DMEM with 10% FetalClone III (HyClone) and penicillin/streptomycin (Sigma-Aldrich) at 37°C as described previously [8]. Cover glasses were treated with 1 mg/ml acidic collagen solution (KOKEN) for 30 min at room temperature, washed twice with ice-cold water, and dried on a clean bench. Collagen coated cover glasses were incubated in PBS for 30 min at 37°C just prior to use. pEGFP constructs were transfected into C2C12 cells and mounted on a confocal laser microscope as for the NG108-15 cells. Images of interference reflection microscopy were obtained by a combination of EYFP excitation and ECFP emission filters.

## Supporting Information

Text S1 Supporting Materials and Methods.
(0.04 MB DOC)

Figure S1 A schematic representation of the domain structure, F-actin-binding activity, and subcellular localization of avian lasp-2 Δe1 fragments. (A) LIM and SH3 indicate the LIM and Src homology 3 domains, respectively. The number of nebulin repeats is shown by n1 to n3. The Δe1 fragment has a defective coding region of exon1 of avian lasp-2 as shown by the double-headed arrow above lasp-2. (B) 0.2 mg/ml G-actin was polymerized with GST-Δe1 (Δe1). Since the expression level of Δe1 in bacteria was low and the purified protein was aggregated easily, we performed a co-precipitation assay of Δe1 at a molar ratio of 1/10. GST-Δe1 (arrowhead in A) was not co-precipitated with actin filament. FA indicates a control experiment using F-actin without the recombinant peptides. The precipitants (p) were separated from the supernatant (s) by ultracentrifugation as described in Materials and Methods. The mobilities of molecular mass markers are listed on the right of the gel images in kilodaltons. (C) Fluorescence image of NG108-15 cells transfected with EGFP-Δe1. The bar represents 10 µm.
(0.11 MB TIF)
