# Supplementary material for: Contribution of the LIM Domain and Nebulin-Repeats to the Interaction of Lasp-2 with Actin Filaments and Focal Adhesions
Source: PLoS One. 2009 Oct 23;4(10):e7530. doi: 10.1371/journal.pone.0007530 (PMC2761545; doi:10.1371/journal.pone.0007530)
Supplement: Text S1 — Supporting Materials and Methods. (0.04 MB DOC) [file pone.0007530.s001.doc]

## Supporting Information Materials and Methods

**Expression constructs.** To construct the N-terminal-tagged molecule, a cDNA fragment encoding the lasp-2 fragment was inserted into the cloning site following a GST or EGFP sequence. The cDNA of the Δe1 fragments were amplified by PCR from plasmids containing full-length lasp-2 cDNA using the following primer sets, which are shown in 5’ to 3’ direction: CGCGGATCCGACAAGTATTGGCATAAAG and GGGGAATTCGGACTAGAGACAAAAAATTAG.
