# Supplementary figures and images for: Contribution of the LIM Domain and Nebulin-Repeats to the Interaction of Lasp-2 with Actin Filaments and Focal Adhesions
Source: PLoS One. 2009 Oct 23;4(10):e7530. doi: 10.1371/journal.pone.0007530 (PMC2761545; doi:10.1371/journal.pone.0007530)

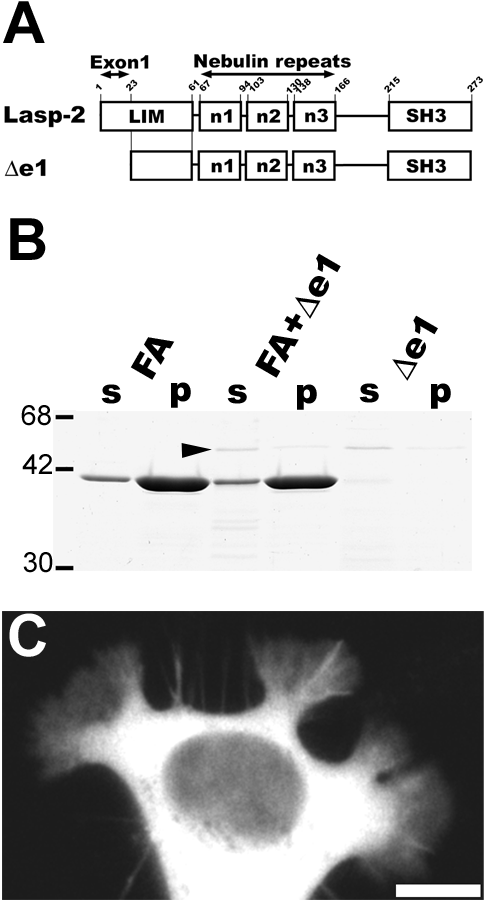

Supplement: Figure S1 — A schematic representation of the domain structure, F-actin-binding activity, and subcellular localization of avian lasp-2 Δe1 fragments. (A) LIM and SH3 indicate the LIM and Src homology 3 domains, respectively. The number of nebulin repeats is shown by n1 to n3. The Δe1 fragment has a defective coding region of exon1 of avian lasp-2 as shown by the double-headed arrow above lasp-2. (B) 0.2 mg/ml G-actin was polymerized with GST-Δe1 (Δe1). Since the expression level of Δe1 in bacteria was low and the purified protein was aggregated easily, we performed a co-precipitation assay of Δe1 at a molar ratio of 1/10. GST-Δe1 (arrowhead in A) was not co-precipitated with actin filament. FA indicates a control experiment using F-actin without the recombinant peptides. The precipitants (p) were separated from the supernatant (s) by ultracentrifugation as described in Materials and Methods. The mobilities of molecular mass markers are listed on the right of the gel images in kilodaltons. (C) Fluorescence image of NG108-15 cells transfected with EGFP-Δe1. The bar represents 10 µm. (0.11 MB TIF) [file pone.0007530.s002.tif]
